# Stereotactic centralized ablative radiation therapy: a framework for ultra-heterogeneous radiotherapy of bulky tumors

**DOI:** 10.3389/fonc.2026.1882750

**Published:** 2026-07-01

**Authors:** Jun Yang, Weisi Yan, Qiuxia Lu, Weihua Qi, Junqi Song, Liangfu Han, Waleed F. Mourad, Jiyuan Yang, Xiaodong Wu

**Affiliations:** 1 Janusyn Oncology Institute, Philadelphia, PA, United States; 2 Department of Radiation Oncology, Foshan Chancheng Hospital, Foshan, Guangdong, China; 3 Department of Radiation Medicine, University of Kentucky, Lexington, KY, United States; 4 Biophysics Research Institute of America, Miami, FL, United States

**Keywords:** bulky tumors, core-to-periphery dose ratio, dose painting, SCART, spatially fractionated radiation therapy, stereotactic radiotherapy, UHRT, ultra-heterogeneous radiation therapy

## Abstract

Bulky tumors remain difficult to treat because their size, irregular geometry, proximity to organs at risk, and heterogeneous microenvironment often conflict with standard radiotherapy objectives. Conventional fractionated radiotherapy prioritizes relatively homogeneous target coverage, whereas stereotactic body radiotherapy (SBRT) is most effective when ablative dose can be delivered safely to the entire lesion. Large or anatomically constrained tumors frequently fit neither model. We propose Stereotactic Centralized Ablative Radiation Therapy (SCART) as a reproducible technical framework for ultra-heterogeneous radiotherapy (UHRT) in selected bulky tumors. SCART defines a central SCART treatment volume (STV) within the gross tumor volume (GTV) and prescribes treatment using a core-to-periphery dose ratio (Rcp). Stereotactic apertures are restricted to the STV at each beam angle or control point, concentrating ablative dose centrally, creating a steep dose gradient through a transitional tumor volume (TTV), and maintaining a constrained low-dose rim at the tumor boundary. In this way, SCART shifts the planning objective from uniform whole-tumor coverage to safe maximal core irradiation while preserving peripheral and peritumoral normal-tissue constraints. The approach is motivated by the spatial organization of bulky tumors, in which hypoxic, radioresistant, and immunosuppressive features are often enriched centrally while the periphery remains more vascular and immunologically active. Early clinical experience suggests technical feasibility in carefully selected patients, but the framework remains hypothesis-generating. Prospective studies are needed to define patient selection, dose ranges, reporting metrics, toxicity, response patterns, and rational combinations with systemic therapy.

## 1 Introduction

Radiotherapy for solid tumors has historically been organized around two dominant planning paradigms. Conventional fractionated radiotherapy and modern intensity-modulated radiotherapy (IMRT) generally seek relatively homogeneous dose coverage across the target, with plan design driven by target delineation and normal-tissue avoidance. SBRT, by contrast, uses stereotactic accuracy and steep dose gradients to deliver ablative dose to the entire lesion, with excellent outcomes in appropriately selected small, well-defined tumors. Bulky, biologically heterogeneous, or anatomically constrained tumors expose the limits of both approaches.

Large tumors often create competing priorities that are difficult to reconcile within existing dose paradigms. Uniform whole-volume irradiation may be constrained by normal-tissue tolerance, especially when targets abut serial organs or critical functional structures. Whole-tumor SBRT may be unsafe or technically impractical when the GTV is large, irregular, mobile, or closely apposed to organs at risk. These limitations are particularly relevant in tumors with resistant central burden, mass-effect symptoms, and complex geometry (1).

The challenge is biological as well as dosimetric. Bulky tumors commonly contain spatially organized microenvironments, including a relatively perfused and radiosensitive periphery and a more hypoxic, radioresistant, immunosuppressive core enriched for clonogenic survival and repair capacity (2–9). This spatial biology motivates a treatment architecture in which dose is deliberately structured rather than incidentally heterogeneous.

Spatially fractionated radiation therapy (SFRT) has challenged the assumption that intratumoral dose heterogeneity is necessarily undesirable. GRID therapy, lattice therapy, and partial-tumor irradiation strategies suggest that selective high-dose regions within bulky tumors may support tumor debulking, normal-tissue preservation, and biological modulation (10, 11). Preclinical evidence also indicates that heterogeneous dose delivery can enhance antitumor immune effects when combined with immune checkpoint inhibition (12). Yet these approaches remain heterogeneous in their own design: target selection, dose distribution, planning method, and biological rationale vary substantially across platforms and institutions. A standardized stereotactic framework for deliberately engineered intratumoral dose heterogeneity is still needed.

**Figure 1:**
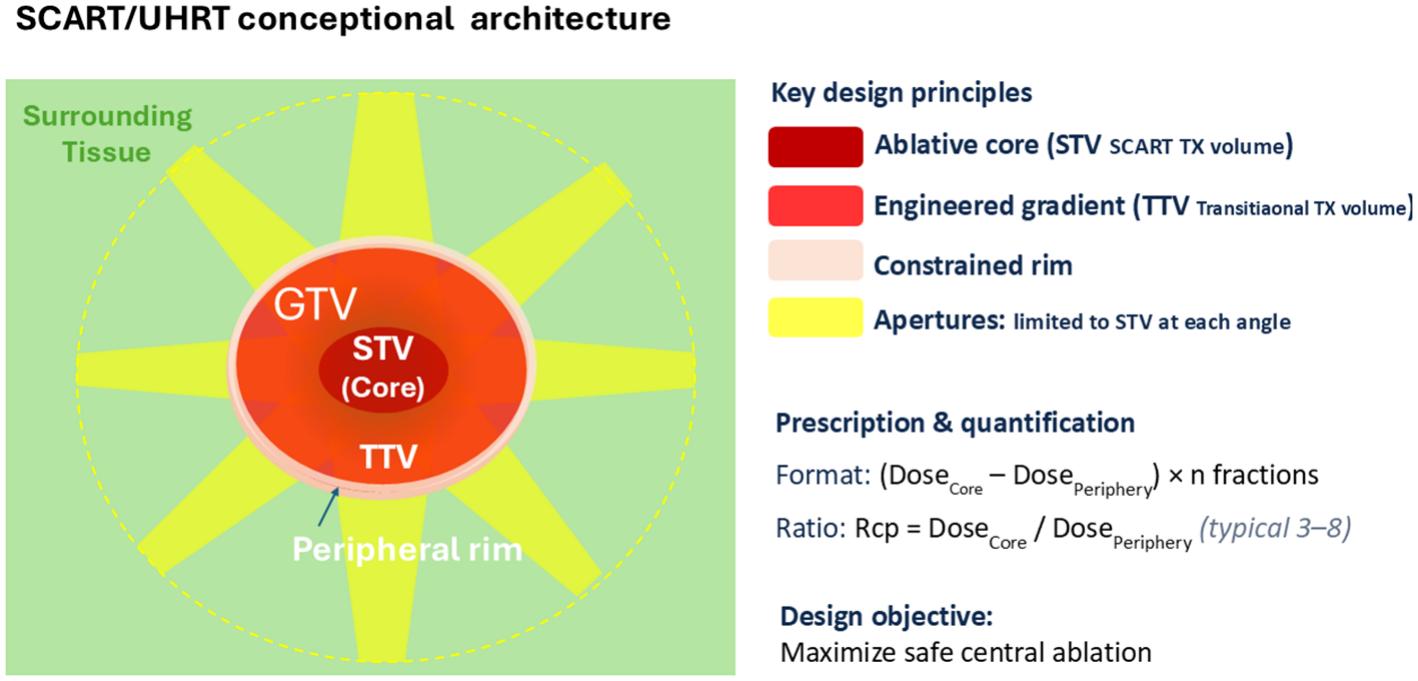
Conceptual architecture of SCART/UHRT. SCART is organized around a centrally located STV receiving ablative dose, a surrounding TTV across which dose decreases rapidly, and a constrained peripheral rim at the tumor boundary. Narrow radiation fields and/or arcs intersect at the STV. In the simplified coplanar geometry, the STV in-plane dimension is approximately dSTV = dGTV/Rcp = dGTV × (DosePeriphery/DoseCore).

SCART is proposed to address that gap. The framework deliberately engineers intratumoral dose heterogeneity by placing an ablative high-dose region within a centrally defined STV, allowing dose to fall steeply through the surrounding tumor, and constraining dose at the tumor boundary. Within this concept, heterogeneity is not a planning imperfection; it is the organizing principle of treatment design, guided by geometric feasibility, normal-tissue safety, and a biological rationale for preferential central intensification (**Figure 1**).

SCART has four defining elements: (i) geometric definition of a central STV within the GTV; (ii) prescription of a core-to-periphery dose ratio (Rcp); (iii) restriction of stereotactic apertures to the STV at every beam angle or control point; and (iv) a peripheral dose limit that maintains a protected low-dose rim at the tumor boundary. Together, these elements concentrate ablative treatment within the tumor core, create a steep internal dose gradient through the TTV, and limit unnecessary high-dose extension into adjacent normal tissues.

**Figure 2:**
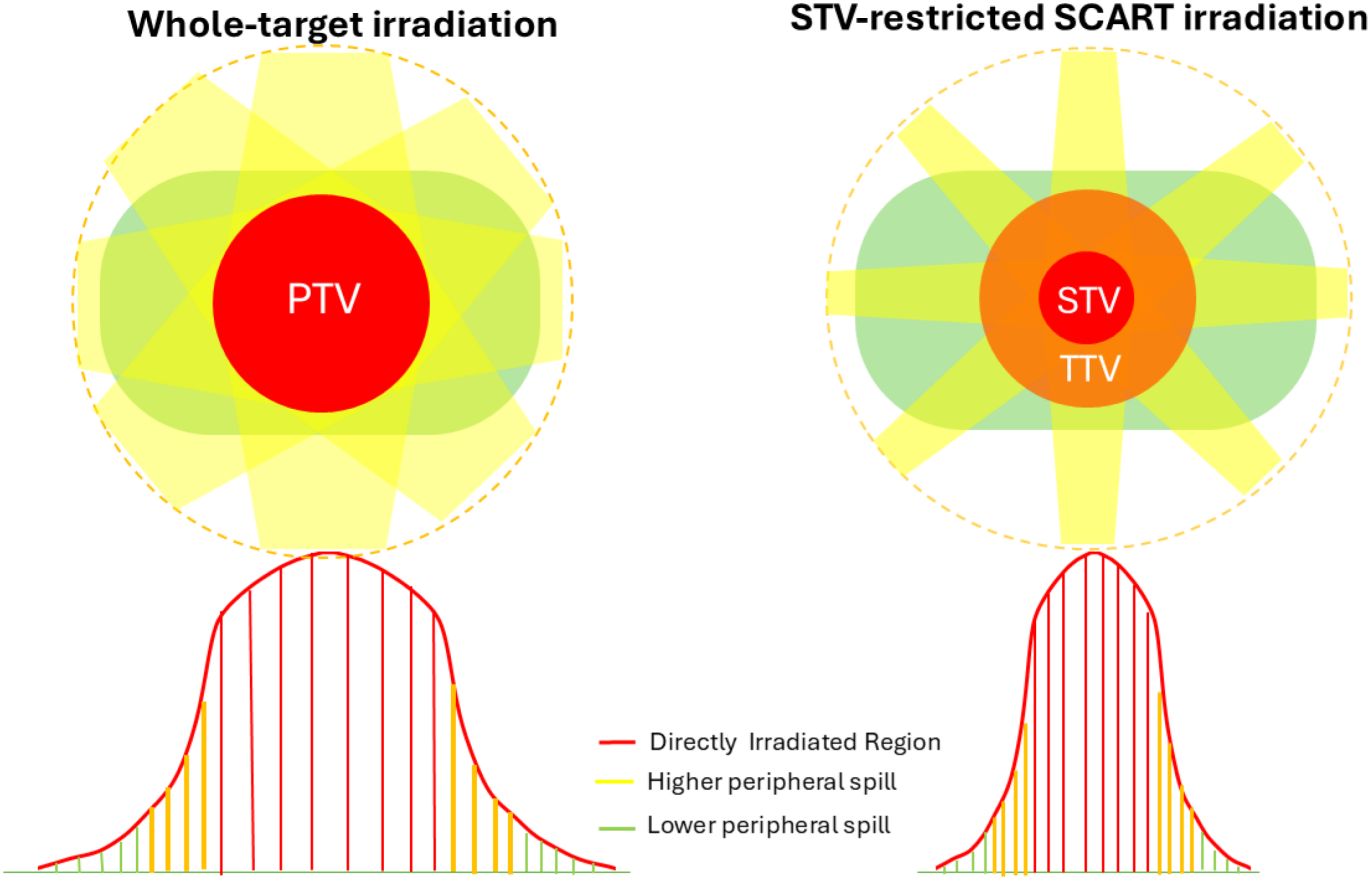
STV-restricted stereotactic delivery and peripheral sparing. In whole-target irradiation strategies, beam apertures conform to the outer target extent, producing high-dose regions that approach the tumor boundary. In SCART, apertures are confined to the central STV, so dose spill traverses tumor tissue before reaching the periphery. This inward displacement of the high-dose region preserves a lower-dose peripheral rim and reduces extension of ablative dose into adjacent normal tissue.

SCART is intended for patients with bulky tumors (typically ≥5 cm in maximum dimension) in whom surgical resection is not feasible and conventional radical radiotherapy is constrained by tumor size, organ-at-risk proximity, or prior radiation exposure (**Figure 2**). Typical clinical scenarios include: (i) locally advanced primary tumors at presentation that are not amenable to radical surgery; (ii) oligoprogressive or oligometastatic bulky lesions during systemic therapy, where local control of the dominant lesion is the immediate clinical objective; (iii) recurrent disease within or adjacent to previously irradiated fields, where full-volume re-irradiation to ablative dose is not tolerable; and (iv) symptomatic bulky lesions where rapid tumor debulking is required. Patients are typically of ECOG performance status 0–2, with adequate baseline organ function and a life expectancy sufficient to derive clinical benefit from durable local control. SCART is not intended for small tumors (<5 cm) amenable to conventional SBRT, for diffuse disease without a dominant bulky lesion, or for patients in whom motion management or immobilization is not feasible.

Here, we describe the rationale, design principles, technical workflow, and early clinical context for SCART in selected bulky tumors. The goal is not to establish clinical superiority over existing modalities, but to define a reproducible framework that can be tested prospectively and compared with conventional IMRT, SBRT, and other spatially heterogeneous radiotherapy strategies.

## 2 The SCART framework

### 2.1 Conceptual architecture: three functional regions

SCART is a stereotactic framework for delivering intentionally ultra-heterogeneous dose within the GTV. It is built on the premise that, in selected bulky or anatomically complex tumors, treatment benefit may not require uniform full-volume ablation. Instead, the plan is organized around three linked goals: concentrate ablative dose in the tumor core, create a controlled rapid fall-off through the surrounding tumor, and limit dose at the tumor periphery.

The tumor is therefore treated as a structured dosimetric volume with three functional regions. The STV is an inward-contracted, centered subvolume of the GTV selected for ablative dosing. Its size is determined by the intended Rcp, tumor geometry, proximity to critical organs, and achievable stereotactic dose gradient. The TTV lies between the STV and the outer GTV boundary; it is not a conventional uniform-coverage shell, but the region through which the engineered dose gradient is expressed. The peripheral rim is likewise not an independent target volume, but the planning consequence of the prescribed boundary dose limit, maintaining a lower-dose interface between tumor and adjacent normal tissue.

Technically, SCART uses narrow fields or control-point apertures confined to the STV. This geometry offers three practical advantages. First, it produces steep intratumoral dose fall-off and permits a central ablative volume while respecting the peripheral dose limit. Second, high-dose spill is contained mainly within the TTV, inside the tumor, rather than extending immediately into surrounding normal tissue. Third, peritumoral normal tissue receives a lower dose than it would under whole-target irradiation of the same bulky lesion.

This design serves both geometric and biological purposes. Geometrically, it may extend stereotactic treatment to lesions that are unsuitable for whole-volume SBRT because of size, shape, motion, or proximity to critical structures. Biologically, it provides a testable framework for aligning physical dose architecture with the core-to-periphery organization commonly observed in bulky tumors. The biological implications remain unproven, but the framework makes the heterogeneity measurable and prospectively studyable.

### 2.2 Prescription format and the core-to-periphery dose ratio

Because SCART specifies both a core ablative dose and a constrained peripheral dose, the prescription should report both values explicitly:


$$\text{S}\text{C}\text{A}\text{R}\text{T}\;\text{p}\text{r}\text{e}\text{s}\text{c}\text{r}\text{i}\text{p}\text{t}\text{i}\text{o}\text{n}:\;\left(\text{D}\text{o}\text{s}{\text{e}}_{\text{C}\text{o}\text{r}\text{e}}–\;\text{D}\text{o}\text{s}{\text{e}}_{\text{P}\text{e}\text{r}\text{i}\text{p}\text{h}\text{e}\text{r}\text{y}}\right)\;\times \;\text{n}\;\text{f}\text{r}\text{a}\text{c}\text{t}\text{i}\text{o}\text{n}\text{s}$$

Intratumoral dose heterogeneity is quantified by the core-to-periphery dose ratio (Rcp):


$$\text{Rcp}=\frac{Dose{\;}_{Core}}{Dose{\;}_{\text{Periphery}}}$$

Rcp is the central planning parameter in SCART. It describes the intended relationship between the ablative central dose and the constrained dose at the tumor edge. Higher Rcp values indicate a steeper central-to-peripheral dose gradient and, for a fixed achievable gradient, a smaller STV. In current early clinical applications, Dose_Core_ has ranged from 15–24 Gy per fraction delivered in 1–3 fractions, and Dose_Periphery_ from 3–5 Gy per fraction, producing Rcp values of approximately 3–8. These values should be interpreted as early-experience ranges rather than mature dose recommendations.

For reproducible prescription and reporting, we recommend the following operational definitions: Dose_Core_ is the dose covering 95% of the STV (D95-STV), consistent with conventional SBRT coverage conventions. Dose_Periphery_ is the maximum dose at the GTV outer boundary, evaluated as Dmax on the GTV surface (or, equivalently, on a thin 2–3 mm shell at the GTV envelope). Rcp is therefore Rcp = D95-STV/Dmax-GTV-surface. We note that other reasonable conventions (e.g. point dose, mean dose within a peripheral rim, D95 or D98 of the rim) may be used provided that the convention is explicitly stated. Standardization of these definitions is a priority for prospective SCART studies and for cross-institutional reporting.

### 2.3 Technical design: principles and planning workflow

We hypothesize that, in bulky tumors, a structured dose distribution that maximizes the ablative-dose volume at the core while preserving the peripheral tumor interface and peritumoral immune compartment may (i) eliminate more clonogens in radioresistant regions and (ii) release more tumor antigens and damage-associated molecular patterns (DAMPs) in a setting where immune processing remains at least partially preserved. SCART operationalizes this hypothesis through three planning principles:

- Normal tissue safety as the primary constraint: The peripheral dose is limited to 3–5 Gy per fraction over 1–3 fractions, a range selected to remain broadly compatible with the tolerance of nearby parallel and serial organs, while recognizing that site-specific constraints must govern final plan approval.
- Ablative dose to the STV: A high stereotactic per-fraction dose is prescribed to the central STV to target radioresistant clonogens and induce substantial tumor-cell injury within the dominant central burden.
- Maximization of the feasible ablative core: Once the peripheral safety constraint and central dose are fixed, the STV size becomes the key adjustable parameter. Steeper intratumoral gradients allow a larger ablative core without violating peripheral dose limits; SCART planning therefore seeks the largest STV supported by the tumor geometry, OAR configuration, motion envelope, and delivery platform.

#### 2.3.1 STV geometry and volume maximization

For a typical coplanar 6-MV volumetric modulated arc therapy (VMAT) plan rotating around the superior–inferior (S–I) axis, the STV may be shaped as a spindle-like central subvolume aligned with the GTV S–I axis and proportionally reduced in the axial plane. Under this simplified geometry, the in-plane STV dimension is primarily determined by the intended Rcp:


$$d_{STV}=\frac{d_{GTV}}{\text{Rcp}}=d_{GTV}\times \frac{Dose{\;}_{Periphery}}{Dose{\;}_{Core}}$$

This expression is an idealized geometric guideline derived for a spherical GTV under coplanar VMAT delivery. In real clinical practice, irregular tumor shape, intra- and inter-fraction motion, tissue heterogeneity, organ-at-risk proximity, beam arrangement, VMAT modulation limits, small-field effects, and platform-specific delivery capabilities may all cause the achievable STV size and dose gradient to deviate from this estimate. The expression should therefore be understood as a starting point for plan design and a conceptual tool for prescription writing, not as a prescriptive clinical planning rule. The final STV is selected after iterative planning that respects OAR constraints, motion management, and platform limitations.

S–I length. Under breath-hold or gating, the STV was contracted 3–5 mm from the GTV in the S–I direction to absorb residual motion uncertainty. Under real-time tracking (CyberKnife), the STV retained the full GTV S–I length. An ITV-based approach for the STV was deliberately avoided, because enlarging the STV to encompass the motion envelope would dilute the central ablative dose and undermine the Rcp design principle; motion is therefore managed at the GTV level via breath-hold, gating, or real-time tracking, with a compensatory S–I contraction of the STV where residual motion uncertainty remains.

If the S–I axis is preserved and only the in-plane dimensions scale with Rcp, the corresponding STV volume scales approximately as:


$$Vol_{STV}\approx \frac{VOL{\;}_{GTV}}{{\text{Rcp}}^{2}}$$

where d_STV_ and d_GTV_ denote the in-plane dimensions of the STV and GTV, respectively. For example, with Dose_Core_ = 20 Gy and Dose_Periphery_ = 5 Gy (Rcp = 4), the in-plane STV dimension is approximately 1/4 of the GTV, corresponding in the coplanar approximation to an STV volume of about 1/16 the GTV. The STV should be placed centrally enough to permit gradient development and preservation of the peripheral rim, while irregular tumors may require an STV that reflects the safest and most practical central ablative region rather than a simple scaled copy of the GTV. Further derivation is provided in **Supplementary S2**.

#### 2.3.2 Aperture restriction as the defining technical feature

After the STV is defined, apertures—whether static fields, MLC-shaped segments, or VMAT control-point apertures—are confined to the STV at every beam angle instead of being expanded to encompass the full GTV. **Supplementary S1** illustrates this geometric principle that smaller apertures generate higher dose gradient. This single planning rule produces the dose architecture that distinguishes SCART from uniform-coverage approaches: the highest dose is concentrated in the tumor core, a steep intratumoral gradient is generated through the TTV, and the tumor edge remains within the prescribed peripheral dose limit.

The steep gradient produced by aperture restriction allows Rcp to function as a planning input rather than a label calculated after the plan is finished. The clinician specifies a core dose and a peripheral dose (which together define Rcp), and the STV is then sized so that the gradient generated by the restricted apertures brings the dose from the core value down to the peripheral value exactly at the GTV boundary. The mapping is one-way and deterministic: a higher Rcp requires a steeper dose drop, which can only develop over a longer fall-off distance, and therefore yields a smaller STV with more intervening tumor tissue between the STV and GTV boundaries; a lower Rcp permits a larger STV with a shorter, less steep fall-off. This STV size is also the maximum ablation volume achievable for the prescribed peripheral dose — a smaller STV under-ablates the tumor, while a larger STV cannot sustain the required gradient against the peripheral dose constraint (**Supplementary S2**). The complete dose architecture is therefore specified by the prescription before any planning is performed.

The approach is naturally suited to small-field stereotactic delivery, which provides penumbra control, conformity to the STV, and steep internal gradients from overlapping beam geometry. Implementation is platform-agnostic: STV-restricted apertures may be realized with non-coplanar static beams, VMAT, robotic stereotactic systems, or other image-guided stereotactic techniques capable of accurate small-field irradiation. The essential requirement is consistent aperture restriction across beam directions, not a specific treatment machine.

#### 2.3.3 Planning workflow, QA, and delivery

A standardized SCART planning process includes four steps:

- Define the GTV and prescribe both a core ablative dose and a clinically acceptable peripheral dose limit, accounting for tumor site, nearby organs at risk, motion, and prior radiation exposure.
- Define the STV according to GTV geometry, intended Rcp, achievable dose gradient, and platform-specific delivery limits, using manual or automated treatment-planning tools.
- Configure the stereotactic beam or arc arrangement so that all apertures remain confined to the STV.
- Optimize and evaluate the plan to maximize dose concentration within the STV while keeping the peripheral GTV dose and all organ-at-risk doses within prescribed constraints.

Plan reporting should extend beyond conventional organ-at-risk metrics to capture the intended internal dose architecture. Recommended parameters include STV volume; STV coverage by the core prescription; maximum dose; GTV coverage by the peripheral dose; Rcp; GTV mean dose; relevant dose-volume metrics for the TTV and peripheral rim; equivalent uniform dose (EUD), when available; and platform-specific motion-management details.

Because SCART relies on stereotactic apertures restricted to the STV, small-field dosimetry uncertainties directly affect both the realized core dose and the planned fall-off through the TTV and rim. SCART plan reporting should therefore include, at minimum: (i) dose calculation algorithm and dose-grid size (typically ≤ 2 mm for STV-relevant dose evaluation); (ii) the smallest field equivalent square used in plan generation and the corresponding output factor measurement protocol with reference to TRS-483 (13); (iii) MLC modeling parameters relevant to small-aperture delivery, including leaf-position accuracy and dosimetric leaf-gap; (iv) patient-specific QA results and pass criteria (e.g., gamma analysis criteria); and (v) any commissioning or QA limitations specific to the smallest fields used. Adherence to TG-155 (14) and MPPG 5.b (15) for commissioning and QA is recommended.

Technical requirements for safe SCART delivery extend conventional SBRT standards because high dose is concentrated in a small intratumoral region with a steep internal gradient. Motion management should be matched to tumor site and the available platform: (i) 4DCT-based motion-envelope assessment is performed at simulation for all sites subject to respiratory or organ motion; (ii) for tumors with motion<5 mm, 4DCT-derived ITV-based planning at the GTV level with free breathing may be appropriate; (iii) for tumors with motion ≥5 mm, breath-hold or respiratory gating is preferred, with the STV contracted 3–5 mm in the superior–inferior direction to absorb residual motion uncertainty as described in Section 2.3.1; (iv) real-time tumor tracking (e.g., CyberKnife) is preferred when available and obviates the need for STV contraction. CBCT immediately before each fraction is required for setup verification, with sub-millimeter to millimeter registration accuracy depending on tumor site. For hypofractionated schedules (1–3 fractions), intrafraction monitoring — kV imaging, surface-guided radiotherapy, or implanted fiducial-based tracking where appropriate — should be used to detect intrafraction motion at the millimeter level, with intervention thresholds defined per institutional policy. Margins at the GTV level should follow site-specific institutional SBRT practice; SCART does not introduce additional margins at the STV level, as the STV is contracted from the GTV rather than expanded toward normal tissue.

Inter-fraction anatomical change in bulky tumors treated with hypofractionated SCART schedules can alter the planned STV/TTV/peripheral-rim relationship. CBCT acquired immediately before each fraction permits assessment of tumor volume, edema, internal anatomy shift, and nearby OAR position. Significant change — for example, ≥ 10% reduction in tumor volume, > 3 mm displacement of a serial OAR within the high-gradient region, or development of substantial new central necrosis — should prompt clinical reassessment and consideration of plan adaptation, ranging from offline re-contouring and re-planning to online adaptive delivery where the platform supports it.

## 3 Early clinical context

At its current stage, SCART should be regarded as an investigational treatment framework supported by technical feasibility and an emerging clinical rationale, not as an established standard of care. The intended clinical setting is a bulky or anatomically constrained tumor in which whole-volume SBRT cannot be delivered safely and conventional fractionated radiotherapy is unlikely to provide rapid or clinically meaningful debulking. Potential scenarios include bulky primary tumors, lesions causing mass effect or organ compromise, tumors close to critical organs, and selected cases in which aggressive local cytoreduction is needed but ablative dose to the full tumor boundary is not achievable. .

**Figure 3:**
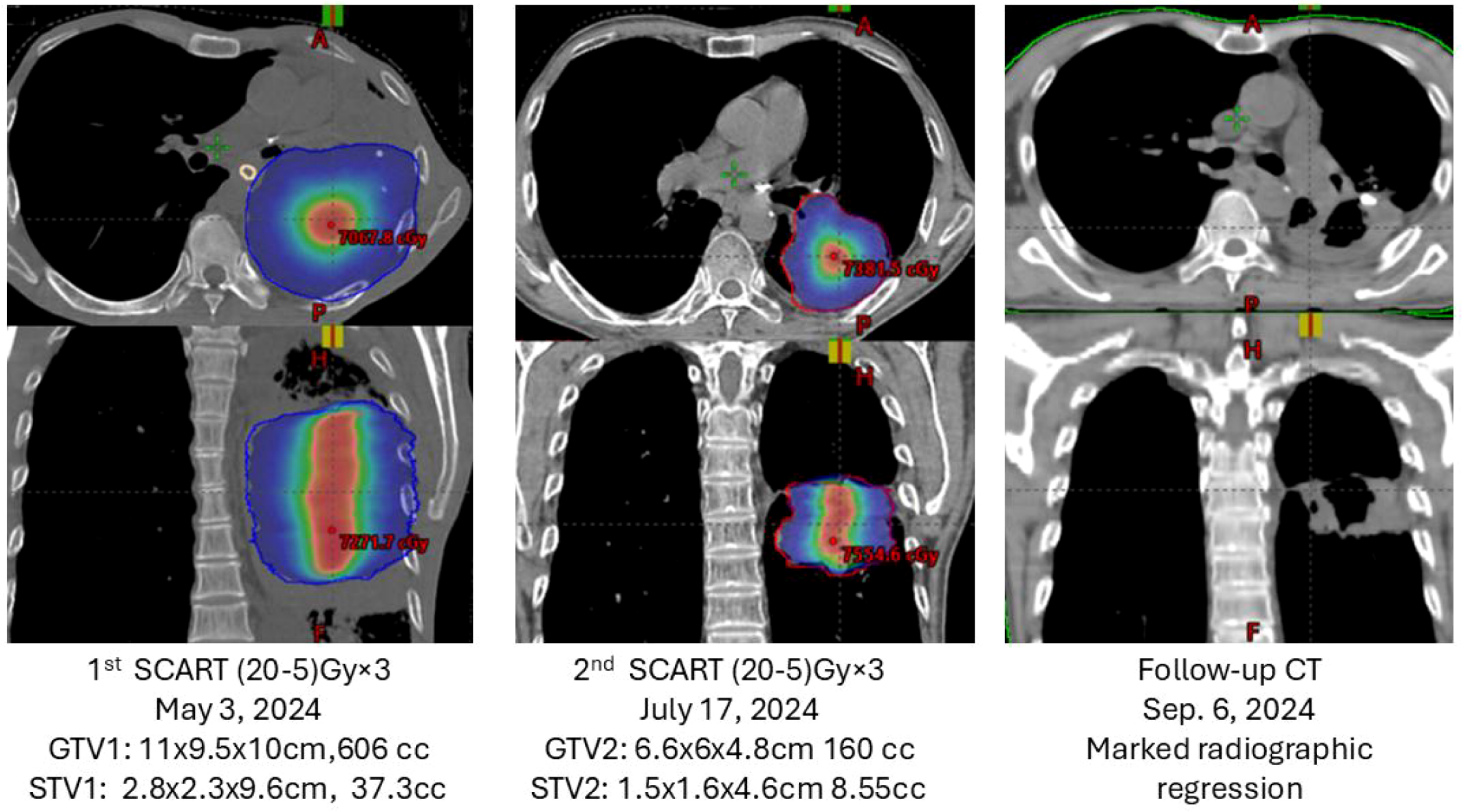
Representative serial clinical implementation of SCART in a bulky lung tumor. A representative patient underwent two sequential SCART courses. The first treatment was delivered on May 3, 2024, with a prescription of (20 – 5) Gy × 3 fractions (Rcp = 4) to a GTV of approximately 606 cm³. The second course was delivered on July 17, 2024, using the same prescription after interval tumor reduction to approximately 160 cm³. Follow-up CT on September 6, 2024, demonstrated marked radiographic regression. This case is shown solely to illustrate technical implementation of the SCART framework in a single patient; it does not constitute evidence of efficacy and should not be interpreted as a clinical outcome. The patient was treated under institutionally approved procedures with informed consent, and all clinical images are de-identified.

**Figure 3** provides a representative serial clinical example in a bulky lung tumor. It illustrates repeated centrally ablative, peripherally constrained planning after interval tumor reduction. The example is included to demonstrate implementation and response pattern within the SCART framework, not to establish efficacy.

The published phase I study (Yang et al. (16)) was designed primarily to evaluate feasibility, dose-limiting toxicity, and the maximum tolerated dose of SCART in a small, clinically heterogeneous cohort. Seventeen patients with 20 bulky unresectable tumors (>5 cm; mean GTV 301 cm³) were enrolled; mean age was 66 years, 70% had stage IV disease, and concurrent systemic therapy (chemotherapy or immunotherapy) was permitted. Treated sites spanned thoracic, abdominal, pelvic, breast, and intracranial locations, and histologies included adenocarcinoma, hepatocellular carcinoma, invasive ductal carcinoma, squamous cell carcinoma, sarcomas, and mesothelioma. Rcp values ranged from approximately 3 to 8. No grade ≥ 3 toxicities were reported, all patients completed treatment, and mean tumor volume decreased from 301 cm³ to 118 cm³; the reported 2-year local control rate of 95% should be interpreted as a preliminary, hypothesis-generating signal of technical feasibility and acceptable short-term tolerance rather than as evidence of comparative efficacy. Cohort heterogeneity, concurrent systemic therapy, small sample size, and limited follow-up (median overall survival 8.94 months) preclude causal attribution of local control to SCART alone. Full per-patient demographics, dosimetric parameters, and outcomes are available in the published report (16). Prospective, standardized studies will be required to characterize efficacy. Other early clinical, case-based, dosimetric, and prospective reports have suggested feasibility in selected bulky tumors and large soft-tissue-forming metastatic lesions, with potential for meaningful debulking and acceptable short-term tolerance (17–19). These observations are encouraging but preliminary and require validation in prospective, site-specific studies with standardized reporting.

The clinical relevance of these early observations lies partly in the symptom burden of bulky tumors. For many patients, decompression, cytoreduction, and relief of local mass effect are important objectives even before long-term local control can be measured. A strategy that produces central tumor injury without requiring ablative dose at the entire tumor edge may therefore have clinical value, provided that safety, patient selection, and integration with systemic therapy are defined prospectively.

## 4 Discussion

### 4.1 Comparison of SCART with IMRT, SBRT, and other spatially heterogeneous approaches

**Figure 4:**
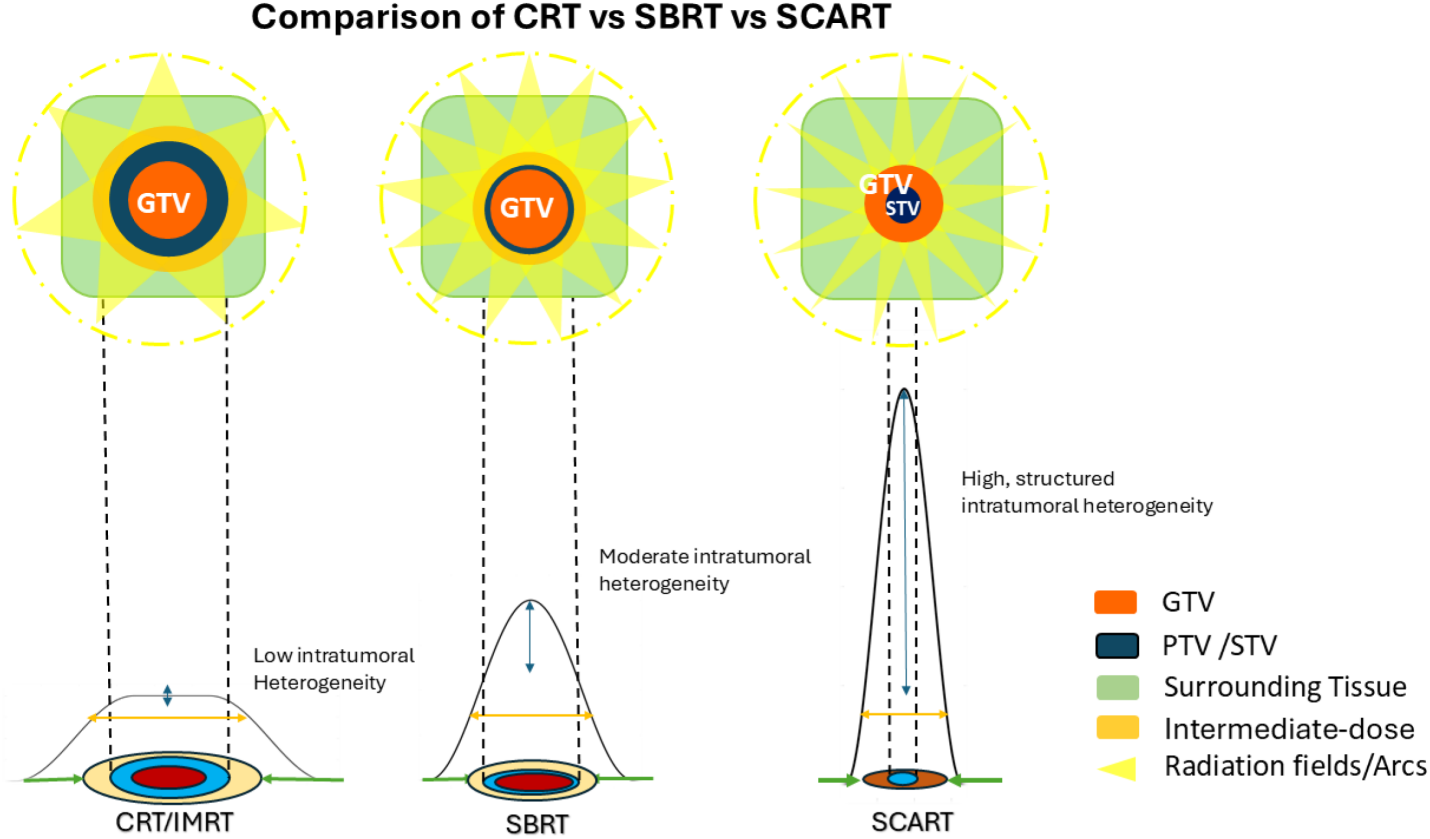
Conceptual comparison of dose architecture in CRT/IMRT, SBRT, and SCART. CRT/IMRT generally pursues relatively homogeneous target coverage. Standard SBRT aims to deliver ablative dose to the entire target, with steep fall-off outside the lesion but high dose extending to the tumor boundary. SCART restricts ablative dose to a central tumor subvolume and deliberately constructs an internal dose gradient toward a constrained peripheral dose. The figure is schematic and does not represent exact dosimetric relationships.

SCART is best understood as a framework for a clinical space not fully addressed by 3D-CRT/IMRT or standard SBRT, rather than as a replacement for existing approaches. Its distinguishing feature is the deliberate organization of dose heterogeneity: the high-dose region, gradient zone, and peripheral dose limit are all defined prospectively. **Figure 4** compares the design philosophies of CRT/IMRT, SBRT, and SCART, emphasizing the shift from uniform coverage or whole-target ablation toward a structured intratumoral dose architecture.

SBRT uses highly conformal, image-guided delivery of ablative dose to the entire target. In selected small tumors, this approach achieves excellent local control with acceptable toxicity. As tumor size, irregularity, motion, or proximity to critical organs increases, however, safe whole-target ablation becomes harder to achieve. SCART preserves key SBRT principles—stereotactic accuracy, steep gradients, small-field delivery, and high-quality image guidance—but changes the target concept: the ablative target is the central STV, while the remaining GTV is incorporated into the TTV and constrained peripheral rim.

SCART also belongs conceptually to the broader family of spatially heterogeneous radiotherapy strategies, including GRID therapy, lattice radiotherapy, microbeam radiotherapy, and partial-tumor approaches such as SBRT-PATHY (10, 11). These approaches share the premise that uniform dose to an entire bulky tumor is not always necessary to achieve clinically meaningful debulking or biological modulation.

SCART differs from these strategies in several specific respects. The high-dose region is a single continuous central subvolume rather than separated strips, vertices, or lattice points. The subvolume is governed by an explicit physical parameter, Rcp, that links prescription, geometry, and dose gradient. The STV is defined geometrically rather than contoured from presumed biological subregions that is defined at. Finally, the defining planning action is aperture restriction to the STV, producing a reproducible internal gradient that can be implemented with standard stereotactic planning tools.

SBRT-PATHY (PArtial Tumor irradiation targeting HYpoxic segment) is the closest conceptual comparator and therefore requires a clear distinction. SBRT-PATHY defines its target biologically, using hypoxia-sensitive PET tracers to identify a hypoxic subvolume within a bulky tumor; the location, shape, and extent of the target therefore depend on functional imaging, and only a small, biologically selected portion of the tumor receives the ablative dose. SCART, in contrast, defines the STV geometrically — the STV’s size and central position are determined by physics factors such as Rcp, tumor geometry, and the achievable stereotactic dose gradient — and its central design goal is fundamentally different: rather than treating a deliberately partial biological subvolume, SCART aims to maximize the ablated volume, i.e. to deliver core ablative doses to the largest contiguous central region of the tumor that is dosimetrically achievable for a given Rcp. This reframes the design question from “which subregion should we treat?” to “how much of the tumor can we ablate while preserving the prescribed core-to-periphery dose gradient?” SCART also offers a specific, reproducible method to realize this design. It is thus a physics-driven structural framework that can be implemented with conventional anatomical imaging, while remaining compatible with future biologically guided refinement (for example, using hypoxia imaging to further optimize STV placement within the GTV).

A further practical advantage of SCART, not shared by any other SFRT modality, is the deterministic relationship between prescription and dose architecture: the geometry of the ablative core is known at the time the prescription is written, because Rcp directly fixes the in-plane scaling dSTV ≈ dGTV/Rcp. A prescription of (20 – 5) Gy × 3 (Rcp = 4) specifies an STV with in-plane dimension approximately one-quarter of the GTV diameter and a coplanar STV volume of roughly one-sixteenth of the GTV; a prescription of (24 – 3) Gy × 3 (Rcp = 8) specifies an STV one-eighth of the GTV in diameter and approximately 1/64 in coplanar volume. By contrast, in GRID therapy and microbeam radiotherapy the size, spacing, and pattern of high-dose regions are determined by collimator hardware; in lattice radiotherapy, by planner-selected vertex templates; in SBRT-PATHY, by hypoxia-sensitive functional imaging. In each of these approaches, the realized dose architecture is a hardware, planning, or imaging output that the prescribing clinician learns about only after the plan has been generated. This geometry-from-prescription property is therefore distinctive to SCART within the SFRT family. It simplifies clinical communication between radiation oncologists, supports consistent reporting across institutions, and makes the framework directly testable in prospective dose-finding studies in which STV size and Rcp can be specified *a priori* rather than reconstructed retrospectively from delivered plans.

### 4.2 Biological implications

Bulky tumors are biologically heterogeneous, with regional differences in oxygenation, vascularity, clonogen density, proliferation, necrosis, stromal composition, and immune interactions (2–9). SCART is designed to align the physical dose distribution with this broad spatial organization. The central STV receives ablative dose directed toward regions likely to contain hypoxic, stress-adapted, radioresistant clonogens and immunosuppressive cellular niches. The surrounding TTV receives a graded sub-ablative dose that may contribute to DNA damage, vascular and stromal remodeling, and partial cytoreduction. The constrained peripheral rim limits high-dose extension into adjacent normal tissues and may help preserve elements of the peritumoral immune microenvironment, including antigen-presenting cells, tumor-infiltrating lymphocytes, and draining lymphatics that could be relevant to systemic immune response (20–26). These biological effects remain hypotheses requiring direct translational testing.

Two qualifications to this biological rationale should be made explicit. First, central hypoxia, radioresistance, and immunosuppression are commonly observed in bulky tumors but are not universal: some tumors may exhibit central necrosis with viable invasive disease near the periphery, irregular or patchy hypoxia patterns, or no clear spatial gradient of the relevant biological features. The geometric STV is therefore a structural approximation of a commonly observed core-to-periphery pattern rather than a biologically tailored target; correspondence between the geometric STV and the actual radioresistant or immunosuppressive subvolume cannot be assumed in any individual patient without functional imaging, spatial pathology, or biomarker characterization. The framework remains agnostic to this biological refinement and is compatible with future biologically guided STV optimization, for example using hypoxia imaging. Second, where the tumor center is in fact hypoxic and partially necrotic — as is often the case in large bulky lesions — direct DNA-damage-mediated cell kill of central cells would be attenuated. The ablative core dose in SCART is therefore not predicated solely on direct cytotoxicity; it is hypothesized to act through vascular ablation and microenvironmental disruption, release of tumor antigens and damage-associated molecular patterns (DAMPs) from the ablated core with consequent immune priming and possible abscopal effects, and mass reduction with reoxygenation of the surviving peripheral rim. From a treatment-planning perspective, liquefactive necrotic regions within the tumor center are not deliberately excluded from the STV: irradiating non-viable, avascular tissue carries no additional toxicity, whereas excluding it would distort the planned dose distribution and reduce the achievable ablated volume — directly contrary to the design goal of maximizing core-volume ablation.

The peripheral zone receiving sub-ablative doses may be insufficient for durable local control as a standalone treatment. Clinically, SCART can be followed by conventional full-tumor irradiation to ensure adequate peripheral dose, or repeated as a staged SCART regimen. SCART is therefore most appropriately positioned as the high-dose component of a combined regimen rather than as a definitive monotherapy for the entire tumor volume.

### 4.3 Limitations

Several limitations should be acknowledged. First, SCART remains early in development; published clinical experience consists mainly of a single-institution phase I study, case-based clinical experience, treatment-planning studies, and early prospective evaluation (16–19). Multi-institutional prospective data are not yet available. Second, geometric and dosimetric accuracy are critical because a very high dose is delivered to a restricted intratumoral region while the tumor edge is constrained; setup uncertainty, respiratory motion, deformable registration error, and platform-specific delivery limits may affect both safety and the intended dose architecture. Third, optimal STV size, dose levels, Rcp ranges, fractionation schedules, and peripheral constraints remain undefined and will likely vary by tumor site and clinical objective. Fourth, not every bulky tumor is suitable for SCART, particularly those with critical organs or tissues embedded within the intended high-dose core, and formal patient-selection criteria remain to be developed. Finally, the proposed biological rationale, especially immune modulation, is hypothesis-generating and requires prospective translational investigation.

Cumulative-dose accounting in the re-irradiation setting requires particular care. SCART’s high per-fraction core dose (typically 15–25 Gy per fraction) makes BED and EQD2-based dose summation less reliable than in conventionally fractionated regimens, because the linear-quadratic model has known limitations at very high per-fraction doses and because tissue recovery between prior radiotherapy and SCART is not well characterized for late-responding normal tissues. We recommend that re-irradiation candidates be evaluated using composite dose summation with deformable image registration where feasible, with explicit acknowledgement of the residual uncertainty in late normal-tissue tolerance after re-irradiation. The peripheral dose constraint of SCART (typically 3–5 Gy per fraction × 1–3 fractions) is intentionally chosen to remain compatible with most realistic cumulative-dose scenarios, but case-by-case evaluation of overlap with prior high-dose volumes, time interval since prior radiotherapy, and the radiosensitivity of nearby late-responding OARs is required.

### 4.4 Future directions

Further development of SCART should proceed along five parallel directions. First, dosimetric standardization is needed for STV definition, peripheral dose limitation, Rcp reporting, and characterization of the TTV and peripheral rim. Second, technical reproducibility should be evaluated across platforms, with attention to small-field dosimetry, image guidance, motion management, and QA. Third, prospective clinical studies should use endpoints matched to SCART’s design intent, including feasibility, acute and late toxicity, symptom relief, radiographic cytoreduction, bridge-to-next-therapy feasibility, and local control. Fourth, translational studies should assess ctDNA dynamics, immune profiling, spatial pathology, and differential response across the STV, TTV, and rim. Fifth, future work should explore rational integration with adaptive radiotherapy, proton or FLASH delivery, and immune checkpoint inhibition.

## 5 Conclusions

SCART/UHRT is a proposed framework for deliberately engineered intratumoral dose heterogeneity in selected bulky tumors. By concentrating ablative dose within the STV, generating steep intratumoral gradients, and enforcing peripheral dose restraint, SCART extends stereotactic principles into clinical scenarios where whole-volume ablation may be unsafe or impractical. The framework is technically implementable with modern image-guided stereotactic platforms, and preliminary experience suggests feasibility and acceptable short-term tolerance. Its potential biological advantages, including interaction with the spatially heterogeneous immune microenvironment, remain unproven and should be evaluated prospectively. SCART should therefore be regarded as an investigational, complementary strategy that warrants technical standardization, biological study, and prospective clinical validation.

## Data Availability

The raw data supporting the conclusions of this article will be made available by the authors, without undue reservation.

## Supplementary material

Supplementary S1 Geometric illustration and derivation of the rapid peripheral dose fall-off produced by STV-restricted stereotactic delivery.

Supplementary S2 Volume-maximization analysis: relationship between R_cp_, achievable dose gradient, and the largest STV compatible with prescribed peripheral dose constraints.
