# Supplementary material for: Stereotactic centralized ablative radiation therapy: a framework for ultra-heterogeneous radiotherapy of bulky tumors
Source: Front Oncol. 2026 Jul 1;16:1882750. doi: 10.3389/fonc.2026.1882750 (PMC13370937; doi:10.3389/fonc.2026.1882750)
Supplement: Supplementary S1 — Geometric illustration and derivation of the rapid peripheral dose fall-off produced by STV-restricted stereotactic delivery. [file DataSheet1.docx]

*Supplementary Material — SCART Framework*

**Supplementary Figure S1**

**Geometric Basis of Dose Gradient Steepness in STV-Restricted Stereotactic Delivery**


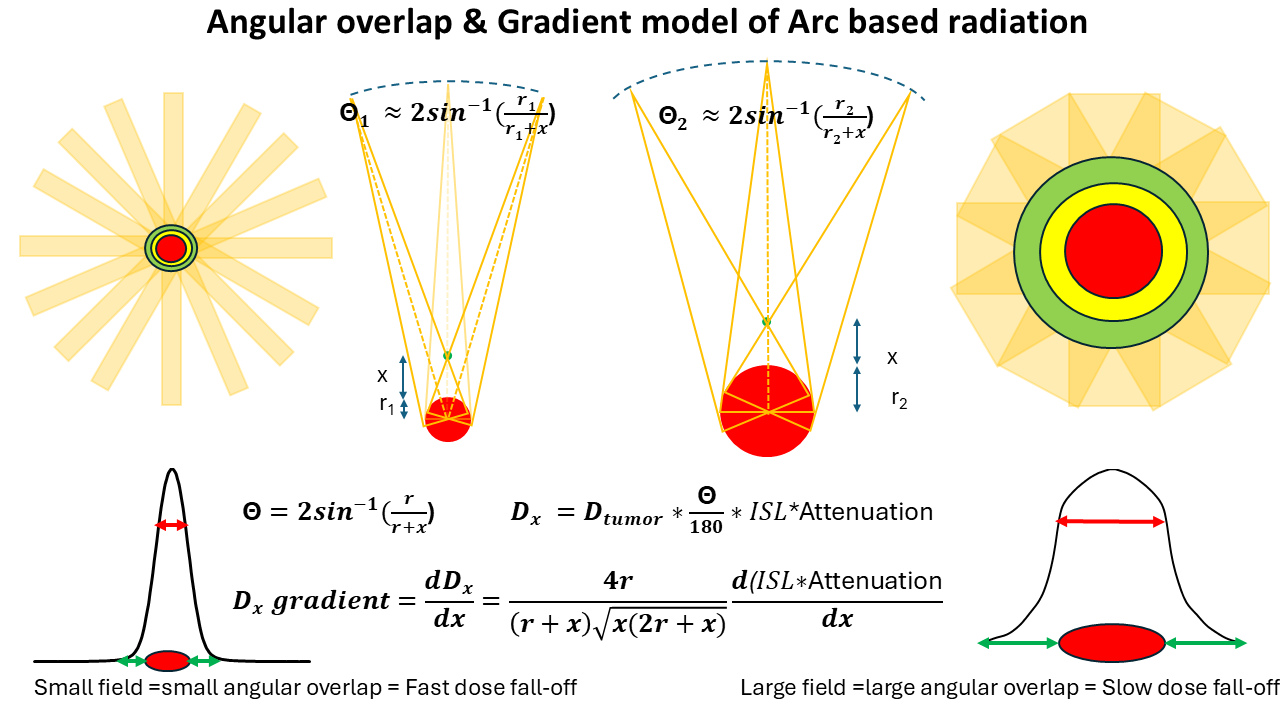


**Figure S1.** Angular overlap and dose gradient model for arc-based stereotactic delivery. Left panel: small field (STV-restricted) geometry with narrow angular overlap (Θ₁) and steep dose fall-off. Right panel: large field (whole-GTV) geometry with broad angular overlap (Θ₂) and slow dose fall-off. Central panel: governing equations for angular subtense, point dose, and dose gradient as a function of target radius r and off-target distance x.

**Explanation**

The rate of dose fall-off outside a stereotactically irradiated target depends critically on the *angular overlap* subtended by the target at points beyond its boundary. For a spherical target of radius *r*, a point located at distance *x* beyond the target surface is subtended by an angular arc:

**Θ ≈ 2sin⁻¹(r / (r + x))**

This angle represents the fraction of delivery directions from which that point receives direct beam contribution. The dose at that point is proportional to Θ, modulated by the inverse-square law and beam attenuation:

**Dₓ = D_tumor_ × (Θ/180) × ISL × Attenuation**

The resulting dose gradient at distance x is:

**dDₓ/dx = [4r / ((r + x)√(x(2r + x)))] × d(ISL × Attenuation)/dx**

Because Θ decreases rapidly with distance for a small target but more slowly for a large one, a small STV generates a steep angular fall-off immediately beyond its boundary, whereas a large target sustains substantial angular overlap — and therefore substantial dose — well beyond its edge.

This geometric relationship provides the physical basis for the steep intratumoral dose gradient in SCART. By restricting beam apertures to the compact STV rather than the full GTV, the angular overlap at points within the surrounding transitional tumor volume (TTV) and at the tumor periphery is sharply reduced, accelerating dose attenuation across the transitional zone and preserving the low-dose peripheral rim that is a defining feature of the framework.

**Supplementary S2**

**Determination of Optimal STV Dimension in SCART**

A central planning question in SCART is how to select an STV size that maximizes the ablated tumor core while remaining within normal-tissue dose constraints. The following analyses use hypothetical spherical tumors to establish a practical geometric rule linking optimal STV dimension to GTV size, prescription dose, and peripheral dose constraint.

**S2.1 Method: Identifying the Optimal STV Dimension**

A fixed SCART prescription of 15 Gy × 3 fractions to the STV and a peripheral tissue constraint of 3 Gy × 3 fractions were applied to a hypothetical spherical tumor of 10 cm diameter. The in-plane STV diameter was increased incrementally by 1 mm, starting from 1 cm. At each increment, the updated STV was used as the exclusive beam-aperture target in VMAT treatment planning, with the maximum MLC opening restricted to that STV. The largest STV achieving adequate ablative coverage without violating the peripheral constraint was defined as the optimal STV dimension.

For the 10 cm tumor under these conditions, the optimal in-plane STV diameter was approximately 2.1 cm, corresponding to 21% of the GTV diameter.

**S2.2 STV/GTV Dimension Ratio Is Independent of Absolute GTV Size**

The same optimization procedure was applied to nine hypothetical spherical tumors ranging from 4 cm to 20 cm in diameter, using the same prescription and peripheral constraint. As shown in Table S1, the optimal STV/GTV dimension ratio was consistent across all tumor sizes, remaining between 20% and 22.5% regardless of absolute GTV diameter. This scale-invariance confirms that the STV/GTV dimension relationship is a property of the dose ratio rather than of absolute tumor size.

**Table S1.** Optimal STV dimension and volume for hypothetical spherical tumors from 4 cm to 20 cm in diameter (prescription: 15 Gy × 3; peripheral constraint: 3 Gy × 3). The STV/GTV dimension ratio is consistent at approximately 21% across all tumor sizes.

| **GTV Diameter (cm)** | **GTV Volume (cc)** | **STV Diameter (cm)** | **STV Volume (cc)** | **STV/GTV Dimension Ratio** | **STV/GTV Volume (%)** |
| --- | --- | --- | --- | --- | --- |
| 4 | 32.8 | 0.9 | 0.9 | 22.5% | 2.7% |
| 6 | 112.1 | 1.2 | 2.8 | 20.0% | 2.5% |
| 8 | 267.0 | 1.8 | 8.3 | 22.5% | 3.1% |
| 10 | 522.6 | 2.1 | 15.0 | 21.0% | 2.9% |
| 12 | 904.0 | 2.5 | 22.5 | 20.8% | 2.5% |
| 14 | 1435.0 | 2.9 | 28.3 | 20.7% | 2.0% |
| 16 | 2143.0 | 3.4 | 47.5 | 21.3% | 2.2% |
| 18 | 3053.0 | 3.9 | 81.8 | 21.7% | 2.7% |
| 20 | 4189.0 | 4.2 | 127.5 | 21.0% | 3.0% |

**S2.3 STV/GTV Ratio Scales with the Peripheral Constraint-to-Prescription Dose Ratio**

The influence of dose parameters on optimal STV size was examined by varying the prescription dose (15–24 Gy per fraction) and peripheral constraint (3 or 5 Gy per fraction) for a fixed 10 cm spherical GTV. As shown in Table S2, the optimal STV/GTV dimension ratio closely tracks the ratio of peripheral constraint dose to prescription dose (D_Periphery_ / D_Core_) across all tested conditions.

This relationship supports a practical design guideline for VMAT-based SCART planning:

**d_stv_ = d_gtv_ × (D_Periphery_ / D_Core_)**

i.e., **d_stv_ = d_gtv_**/Rcp

where *d*_stv_ and *d*_gtv_ are the characteristic in-plane dimensions of the STV and GTV respectively, and Rcp is the core-to-periphery dose ratio. This is equivalent to the geometric relationship described in the main text (Eq. 1), and confirms that the STV selection rule is derivable from first principles rather than being empirically arbitrary.

**Table S2.** Optimal STV/GTV dimension ratio at escalating prescription doses (15–24 Gy per fraction) and two peripheral constraints (3 or 5 Gy per fraction) for a 10 cm spherical GTV. The observed STV/GTV dimension closely matches the Dₚₑʳᵊ/Dᴼᵒʳᵉ ratio across all conditions.

| **Level** | **Prescription Dose** | **Peripheral Constraint** | **Dose_Periphery_ /Dose_Core_ Ratio** | **STV/GTV Dimension** | **STV/GTV Volume (%)** |
| --- | --- | --- | --- | --- | --- |
| −1 | 15 Gy × 1 | 3 Gy × 1 | **20%** | **21%** | 3.0% |
| −1 | 15 Gy × 1 | 5 Gy × 1 | **33%** | **36%** | 10.6% |
| 1 | 15 Gy × 3 | 5 Gy × 3 | **33%** | **36%** | 10.6% |
| 2 | 18 Gy × 3 | 5 Gy × 3 | **28%** | **27%** | 6.3% |
| 3 | 21 Gy × 3 | 5 Gy × 3 | **24%** | **24%** | 4.5% |
| 4 | 24 Gy × 3 | 5 Gy × 3 | **21%** | **21%** | 3.3% |

**S2.4 Summary and Clinical Implications**

Taken together, these analyses demonstrate three properties of the SCART STV selection rule: (1) for a given dose prescription and peripheral constraint, the optimal STV/GTV dimension ratio is approximately constant across a clinically relevant range of tumor sizes (4–20 cm); (2) the ratio is determined by the core-to-periphery dose ratio Rᶜₚ = D_Core_/D_Periphery_ and can be computed directly from the intended prescription; and (3) the corresponding STV volume fraction scales approximately as (D_Periphery_ / D_Core_)², reflecting the quadratic relationship between linear dimension and cross-sectional area.

In clinical practice, these relationships support a reproducible and standardized workflow for STV definition: given a chosen prescription dose and acceptable peripheral constraint, the STV in-plane dimension can be estimated directly as d_gtv_ / Rcp, without iterative trial-and-error. This geometric predictability is a key element of SCART’s identity as a standardized framework that maximizes the volume ablated within normal-tissue safety limits.

*End of Supplementary Material*
